# Removal of toxic hexavalent chromium *via* graphene oxide nanoparticles: study of kinetics, isotherms, and thermodynamics

**DOI:** 10.1039/d4ra03697b

**Published:** 2024-08-05

**Authors:** Zohor Khdoor, Sami Makharza, Mohannad Qurie, Firas Fohely, Abdallah Abu Taha, Silke Hampel

**Affiliations:** a Faculty of Science and Technology, Department of Chemistry, Hebron University P. O. Box 40 Hebron West Bank Palestine; b College of Medicine, Hebron University P. O. Box 40 Hebron West Bank Palestine Makharza.sami@gmail.com; c Department of Earth and Environment Sciences, Faculty of Science and Technology, Al-Quds University Palestine; d Department of Medical Imaging, Faculty of Pharmacy and Medical Science, Hebron University P. O. Box 40 Hebron Palestine; e Department of Biology and Biochemistry, Birzeit University Birzeit Palestine; f IFW Dresden Germany

## Abstract

In this study, graphene oxide (GO) was prepared by the Hummers' method from graphite material. The adsorption potential of GO-200 nm for the removal of Cr(vi) ions was investigated. Fourier transform infrared (FTIR) spectroscopy was used to analyze Cr(vi) before and after adsorption. The adsorption isotherm was fitted by the Langmuir model and the maximum adsorption capacity of the GO was 41.27 mg g^−1^ at 25 °C. Thermodynamic parameters (Δ*G*°), (Δ*H*°), and (Δ*S*°) were calculated and exhibited as +2.63 kJ mol^−1^ K^−1^, +4.30 kJ mol^−1^ K^−1^, and +5.56 kJ mol^−1^ K^−1^ at 30 mg L^−1^ of Cr(vi) solution, respectively.

## Abstract 2

In this study, graphene oxide (GO) was prepared by the Hummers' method from graphite material.

## Graphical abstract

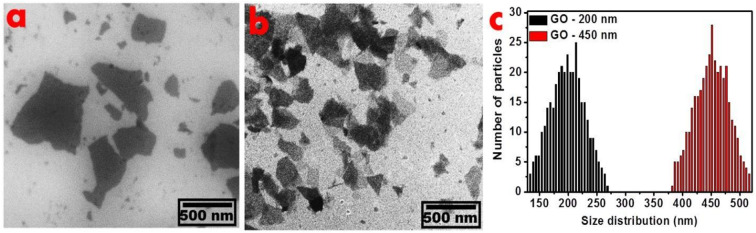

## Introduction

Heavy metal pollution is a major concern of aquatic ecosystems worldwide, even at low levels of exposure. For instance, copper, zinc, cadmium, lead, mercury, arsenic, and chromium metal ions are highly toxic to living organisms due to their persistence, bioaccumulation, non-biodegradability, and environmental stability.^1^ Chromium is commonly found in the environment in Cr(iii) and Cr(vi) oxidation states, which have quite different chemical properties. Cr(iii) is chemically converted to Cr(vi) by redox reaction under certain conditions. Cr(vi) is considered a carcinogenic and mutagenic material.^2^ Several methods have been applied to remove Cr(vi) from aqueous solutions. Among these methods, adsorption is the most promising and effective method for Cr(vi) removal due to its simplicity, cost-effectiveness, applicability for the industry and being eco-friendly.^3^

In this regard, various adsorbents such as biological materials, chitosan, industrial wastes, zeolites, dendrimers, biochar, imprinted materials and activated carbon have been proposed to remove hexavalent chromium Cr(vi) from the water^4–7^ ecosystem. Recently, GO nanoparticles have been introduced as nanoadsorbents, which have drawn additional attention due to their properties such as extremely high surface area and adsorption site, tunable morphology, and much lower intra-particle diffusion distance. These materials do not require high operation and maintenance costs.^2,4,5^ Nanomaterials such as GO are effective in the removal of heavy metals from wastewater, and they are a viable alternative to conventional adsorbents. Among other advantages, GO has received considerable attention due to its unique chemical and physical properties such as hydrophilicity and stability in solution. The abundant oxygen groups such as –OH, –COOH and –C=O distributed on their surfaces imparted during the oxidation of graphite. GO nanoparticles are successfully prepared in our chemical lab^8^ for using as an adsorbent for the removal of hexavalent chromium Cr(vi) from an aqueous solution.

**Scheme 1:**
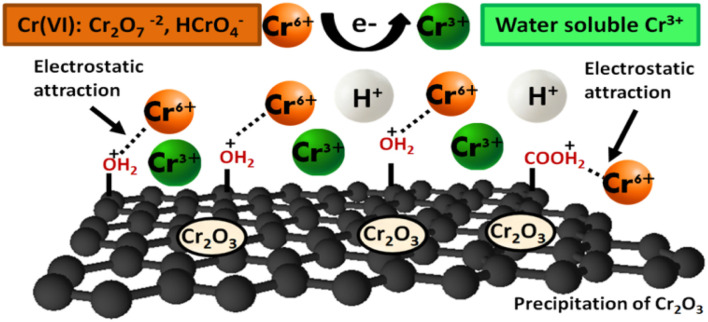
Proposed mechanism for Cr(vi) removal by GO in an acidic environment.

In this work, the adsorptive removal of Cr(vi) metal ions using GO as an adsorbent under different experimental conditions was elucidated. Scheme 1 exhibits the proposed mechanism of reduction of hexavalent Cr to trivalent in acidic conditions. The mechanism for the removal of Cr(vi) using GO includes adsorption through electrostatic attractions,^9^ reduction of Cr(vi) to Cr(iii),^10^ and a probable coordination between chromium ions and ligands.

## Materials and methods

### Preparation of graphite oxide

**Scheme 2:**
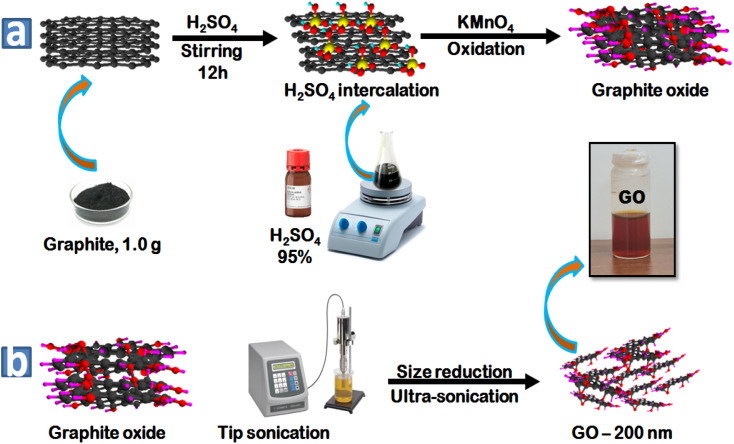
Oxidation of graphite (a) for the preparation of graphite oxide using Hummers' method and (b) size reduction of GO particles under controlled conditions.

Graphite oxide was produced from natural graphite using the modified Hummers' method.^8,11–13^ In Scheme 2, 1.0 g graphite was ground with 50.0 g of NaCl for a few minutes to exfoliate the graphite particles and reduce their dimensions. The ground graphite was added to warm water and collected using filter paper by suction filtration. The dried graphite was mixed with 20 mL H_2_SO_4_ overnight, and the obtained solution was stirred in an ice bath for 45 min and 3 g KMnO_4_ was slowly added as an oxidizing material. After the complete addition of the oxidizer, the mixture was stirred for 30 min at 35 °C, the temperature was raised up to 50 °C for 45 min. Thereafter, 46 mL of distilled water was added gradually to the solution and the solution was kept stirring for 45 min at 98 °C. Subsequently, 140 mL distilled water and 10 mL of 3% H_2_O_2_ were added to the mixture.

The collected sample was filtered and washed three times with 5% HCl and distilled water to remove any of the side products. Finally, the graphite oxide powder was obtained after drying in a vacuum at 30 °C for 24 h.

### Synthesis of GO-200 nm

After purification of graphite oxide by centrifugation, the GO-200 nm nanoparticles were prepared according to our previous protocol.^8^ Subsequently, 1.0 mg mL^−1^ of graphite oxide was sonicated in an ultra-sonication bath under controlled conditions (Scheme 2b).

### Batch adsorption experiments

The batch adsorption experiments were used to study the effect of pH at the range 2.0 to 7.0, mass of adsorbate (1, 5, 10, 20, 40, 60, 100 and 140 mg), time (1, 20, 40, 80, 160, and 240 min), temperature in the range of 25–55 °C, and Cr(vi) initial concentration (30, 50, 100, 200, 300, 400 and 500 mg L^−1^). Further experiments were performed to characterize the adsorption kinetics, isotherms, and thermodynamics at the optimum values of pH and mass of graphene oxide. Chromium(vi) stock solution (1000 ppm) was prepared by dissolving 0.283 g of potassium dichromate (K_2_Cr_2_O_7_) in 100 mL distilled water. Analytical solutions were prepared from the stock solution by using a dilution factor. The adsorption experiments were performed in 25 mL flasks containing 20 mL of a series of Cr(vi) solutions. The pH of the solution was adjusted to 3.0 and 2 g L^−1^ of graphene oxide material was added. The mixture was sonicated to homogenize the mixture.

After 24 h of incubation, the mixture was filtered using a syringe filter nylon with pore size (0.45 μm) and stored at 4 °C. The residual total chromium concentration (Cr(vi) + Cr(iii)) was analysed by atomic adsorption spectroscopy, while the residual Cr(vi) was analysed using a UV-visible spectrophotometer which was assessed by 1,5-diphenylcarbazide method, the absorbance of the red-violet coloured solution was obtained from the reaction after 10 min at 540 nm.^14^ The adsorption capacity [*q*_e_ (mg g^−1^)] and percentage removal efficiency of Cr(vi) were calculated using eqn (1) and (2):^15^1
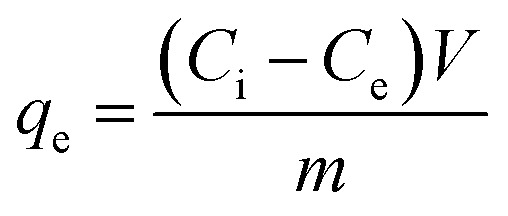
2
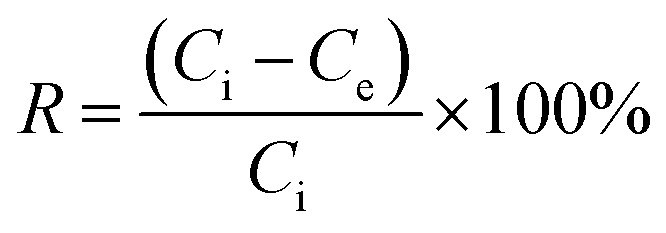


### Variation parameters

The kinetic study was carried out at different time intervals (1, 20, 40, 80, 160 and 240 min) in separate experiments for 50 and 100 mg L^−1^ of Cr(vi) solution. The variation of initial Cr(vi) concentrations (30, 50, 100, 200, 300, 400 and 500 mg L^−1^) and isotherm models were employed in this study.

## Results and discussion

### Scanning electron microscopy (SEM)

**Fig. 1:**
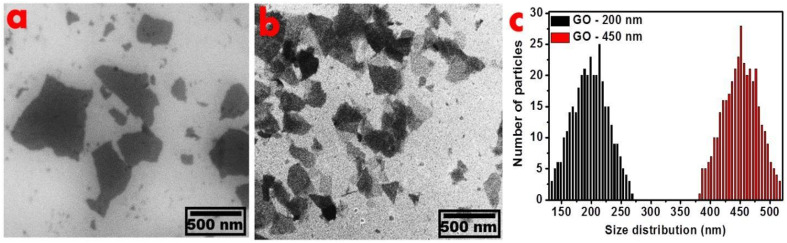
SEM images of (a) GO-450 nm and (b) GO-200 nm and (c) is the average width (nm) of GO particles deduced from the SEM image, the size distribution of GO-450 is ±35 nm, GO-200 is ±20 nm.

The lateral sizes of the GO particles were elucidated by scanning electron microscopy, as shown in Fig. 1. The as-prepared graphite oxide is presented in panel (a). The GO-200 nm with the reduced size after sonication under controlled conditions is presented in panel (b).

Panel (c) exhibits the statistical analyses of particles deduced from SEM images. According to our literature reports,^8,11,12^ the as-prepared GO particles exhibited 450 nm lateral size distribution. The as-prepared GO particles were treated under hard sonication to increase the surface-to-volume ratio. In panel (c), the number of GO particles is approximately 250 to measure the size distribution of samples.

### FTIR spectra of GO and GO–Cr(vi) system

**Fig. 2:**
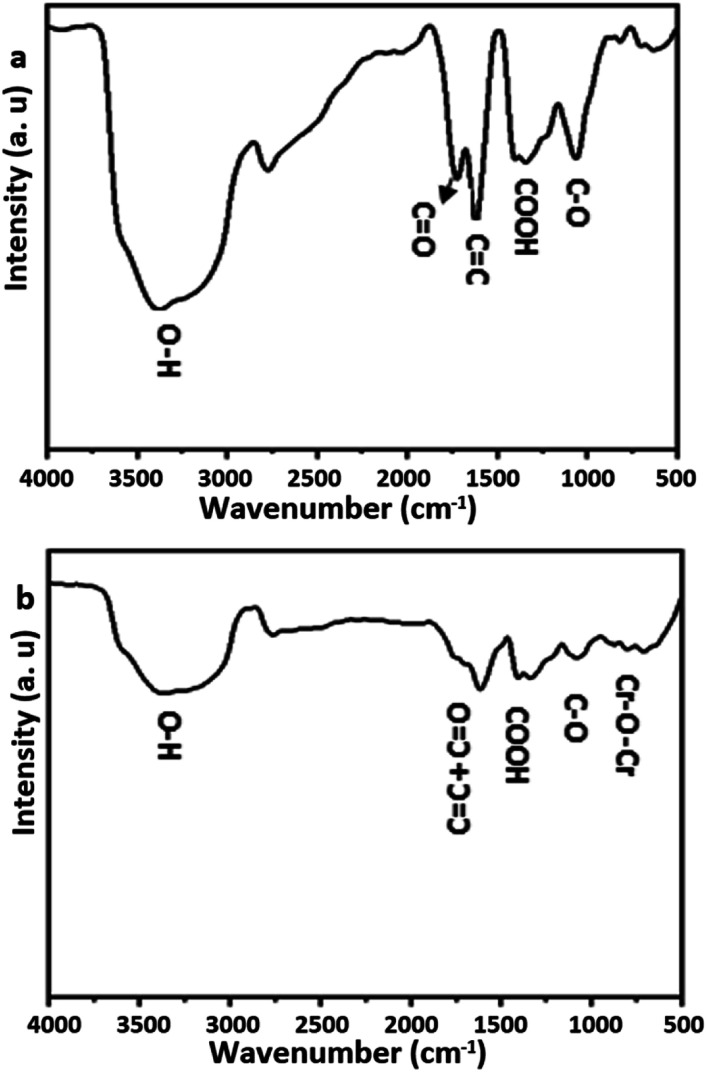
FTIR spectra of (a) graphene oxide (b) graphene oxide treated Cr(vi).

The characteristic peaks of pristine GO-200 nm and GO/Cr(vi) are shown in Fig. 2. As shown in panel (a), the GO revealed the main functional groups distributed on the surface and the edges of GO particles. The peak position of the hydroxyl (–OH) group appears at 3365 cm^−1^ stretching vibration, and the carbonyl (C=O) group at 1731 cm^−1^. The carbon-to-carbon double bond (C=C) takes position at 1619 cm^−1^, this functional group represents the sp^2^ regime of the 2D graphene layer. The epoxy (C–O) group becomes visible in the lower frequency region at 1400 cm^−1^.^8,16^ The FTIR spectrum of GO–Cr(vi) nano-system was performed as shown in Fig. 2b. The GO–Cr(vi) exhibits three band positions at 715, 804 and 890 cm^−1^, which are assigned to Cr=O and Cr–O–Cr bonds, indicating that Cr(vi) was adsorbed on the surface of GO. Furthermore, the normalized peak intensities were reduced in high and low-frequency regions. A subtle shift in the absorption peaks was observed, which was assigned to the perturbation of energy due to the new coordination between the oxygen groups and chromium ions. These bands are usually shifted to lower or higher frequencies.^17,18^

### Adsorption studies: effect of pH

The pH is a key parameter controlling the Cr(vi) adsorption process. It has a significant effect on the surface charge, binding sites of the adsorbent and metal ion speciation. There are several anionic forms of Cr(vi) existing in the solution, such as CrO_4_^2−^, dichromate (Cr_2_O_7_^2−^) and hydrogen chromate (HCrO_4_^−^). At 2 ≤ pH ≤ 6, it exists in two equilibrium forms of (Cr_2_O_7_^2−^) and (HCrO^4−^), however, chromate anion (CrO_4_^2−^) predominates under alkaline conditions.^15^ The initial concentration of chromium ions is 50 mg L^−1^ with 2 h of contact time and 25 °C.

### Percent removal and adsorption capacity

**Fig. 3:**
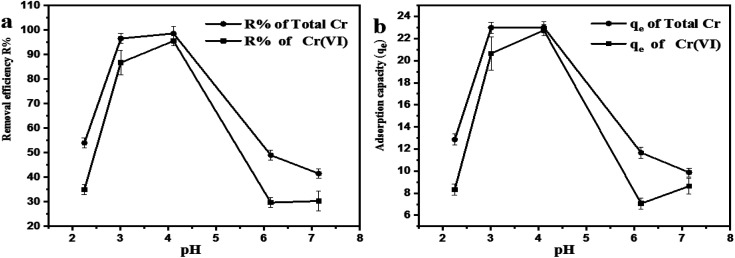
Effect of adjusted pH on (a) percentage removal and (b) adsorption capacity (*q*_e_).

Fig. 3 reveals the percent removal and adsorption capacity (*q*_e_) of Cr(vi) adsorbed onto the basal plane of GO particles as a function of pH. The result indicated that the pH between 3 to 4 has the highest percentage removal of the total chromium and Cr(vi). Cr(vi) is partially reduced to Cr(iii) by the reductive surface hydroxyl groups on the surfaces of GO.^10^ This reaction catalyzed by electrons might be caused by the electrons on the carbocyclic six-membered ring of GO.^17^ The resulting Cr(iii) is either released back into the solution at lower pH in the form of water-soluble Cr(iii) species or precipitated as Cr_2_O_3_ to achieve the performance of adsorption.^10^

### Kinetic studies

#### Contact time

Generally, the removal of chromium ions increases as the contact time increases until the equilibrium is reached. Once the equilibrium is reached, the adsorption process of metal ions becomes constant. At the beginning of the adsorption process, a large number of active sites are available for the adsorbate and the process proceeds very fast, however, as the active sites are filled, the adsorption proceeds slowly until the equilibrium is reached.^9^

**Fig. 4:**
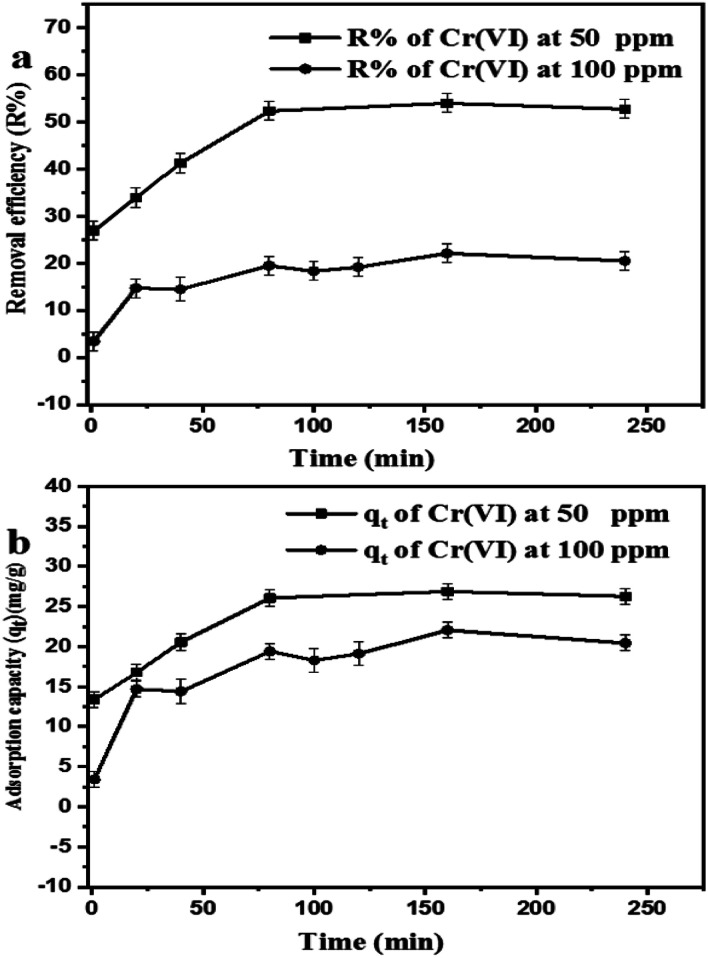
Effect of the contact time on (a) percentage removal efficiency (*R*%) and (b) adsorption capacity (*q*_*t*_) of Cr(vi).

Fig. 4 summarizes the effect of the contact time on the percent removal and adsorption capacity of chromium ions by GO nanoparticles. The percentage removal was increased during the first 80 min, and then it reached a plateau at equilibrium.

#### Kinetic models of adsorption

The Pseudo-first order, pseudo-second order, intra-particle diffusion and Elovich kinetic models have been investigated in this study. These models explain the mechanisms that control the adsorption processes. The following linear forms expressed the pseudo-first-order (eqn (3)),^15^ pseudo-second-order (eqn (4)), intra-particle diffusion model (eqn (5)), and Elovich kinetic model in (eqn (6)).^19^3
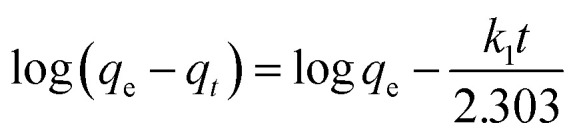
4
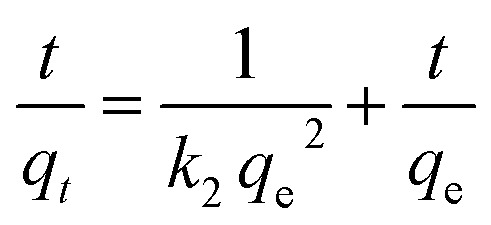
5
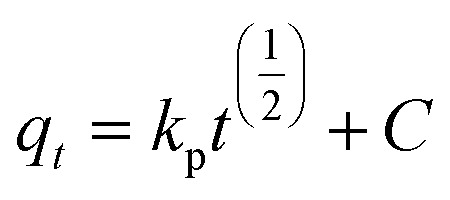
6
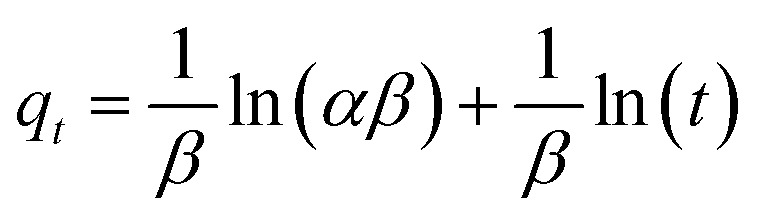
where *q*_e_ and *q*_*t*_ are the adsorption capacities (mg g^−1^) at equilibrium and at a time (*t*) respectively, *k*_2_ is the rate constant of second-order adsorption (g mg^−1^ min^−1^), *k*_1_ is the pseudo-first-order rate constant (min^−1^), *k*_p_ is the rate constant of intra-particle diffusion (mg g^−1^ min^−1/2^), *C* is the intercept represents the thickness of the boundary layer, *α* is the initial adsorption rate (mg min^−1^), *β* is the extent of surface coverage and activated energy (g mg^−1^).

**Fig. 5:**
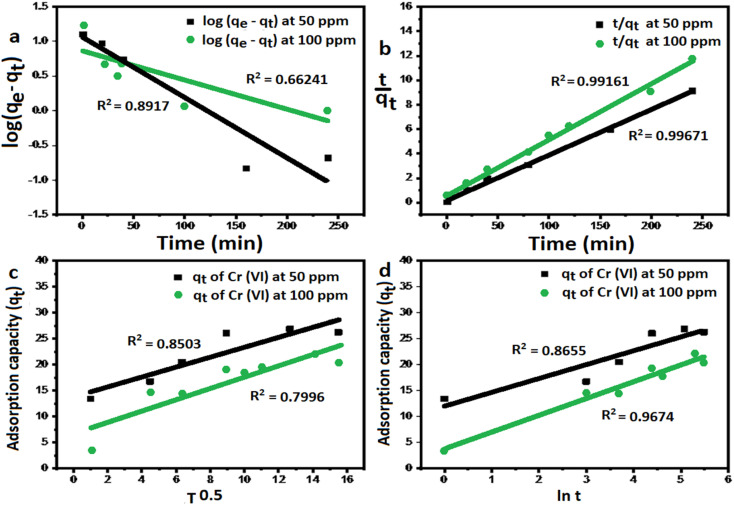
The kinetic models of Cr(vi) adsorption using GO-200 nm. (a) Pseudo first order model, (b) pseudo-second order model, (c) intra-particle diffusion model, and (d) Elovich kinetic model.

Fig. 5a shows the pseudo-first-order model with linear regression correlation coefficient (*R*^2^) and describes the kinetics of Cr(vi) adsorption onto GO nanoparticles. The results support the assumption that adsorption is chemisorption and related to valence forces through the sharing or exchange of electrons between the GO and Cr(vi).^15,19^

The rate constant of the pseudo-first-order kinetic was found to be decreased with increasing initial Cr(vi) concentration,^17^ indicating that the adsorption of Cr(vi) onto GO would be faster at a lower initial concentration.

Fig. 5b exhibits a linear relation with high correlation coefficient for 50 and 100 ppm, which reflects a very strong correlation between the parameters and a good fitting of the experimental data with pseudo second order kinetic model, this is supported by the agreement between the theoretical values and the experimental values and data are listed in Table 1.

The kinetic models with different correlation coefficients at 50, 100 mg L^−1^

**Table:** 

Conc. (mg L^−1^)	Pseudo second-order model				Pseudo first-order model			
	*R* ^2^	*q*_e (exp)_	*q*_e (cal)_	*k*_2_	*R* ^2^	*q*_e (exp)_	*q*_e (cal)_	*k*_1_
50	0.9967	26.06	27.38	4.96 × 10^−3^	0.8917	26.06	11.195	0.0197
100	0.9916	19.39	21.97	3.55 × 10^−3^	0.6624	19.39	7.391	0.0098

**Table:** 

Conc. (mg L^−1^)	Intra-particle model			Elovich model		
	*R* ^2^	*k*_p_	*C*	*R* ^2^	*α*	*β*
50	0.8503	0.960	13.66	0.9674	245.4	0.375
100	0.7996	1.086	6.79	0.8655	10.19	0.308

#### Adsorption isotherm models

**Fig. 6:**
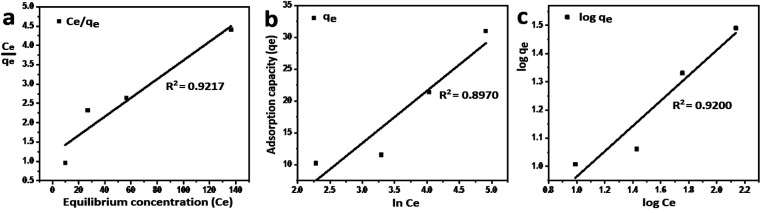
Equilibrium studies of Cr(vi) adsorption by GO, (a) Langmuir isotherm model, (b) Temkin isotherm model, and (c) Freundlich isotherm model.

Among various adsorption isotherms, Langmuir, Freundlich and Temkin models were applied in this study to understand the adsorption behaviour of Cr(vi) ions by GO particles, which is observed in Fig. 6. The linear forms of Langmuir, Freundlich, and Temkin are expressed in eqn (7)–(9), respectively.7
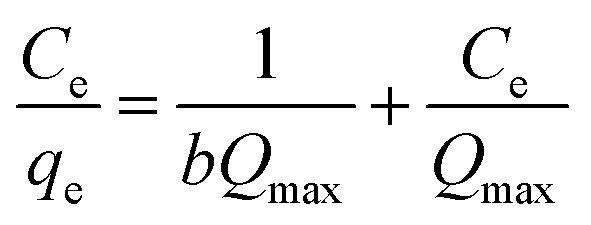
8
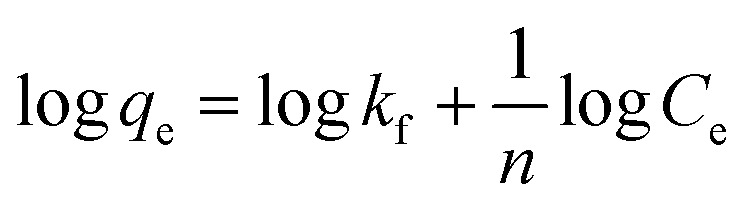

9 *q*_e_ = *B*_T_ ln *k*_T_ + *B*_T_ ln *C*_e_

where *C*_e_ refers to the equilibrium concentration of the remaining solute in the solution (mg L^−1^), *q*_e_ is the amount of solute adsorbed per unit mass of the adsorbent at equilibrium (mg g^−1^), *Q*_max_ is the amount of adsorbate per unit mass of the adsorbent at complete monolayer coverage (mmol g^−1^), *b* is a Langmuir constant. The variables (*n*) and (*k*_f_) are Freundlich constants that are related to the adsorption intensity and adsorption capacity, respectively, 1/*n* represents the heterogeneity factor. The *B*_T_ is the constant related to the heat of sorption (J mol^−1^), and *k*_T_ is the Temkin isotherm constant. Fig. 6 indicates that the adsorption of Cr(vi) ions by GO is well described by Langmuir isotherm parameters that are listed in Table 2. The calculated *Q*_max_ is 41.27 mg g^−1^ for the adsorption by GO.

**Table:** Isotherm models parameters for adsorption of Cr(vi) by GO

Isotherm models	*R* ^2^	Parameters	
Langmuir	0.9217	*Q*_max_ = 41.27 mg g^−1^	*b* = 0.02035 mg^−1^
Temkin	0.8970	*B*_T_ = 8.226	*k*_T_ = 0.254
Freundlich	0.9200	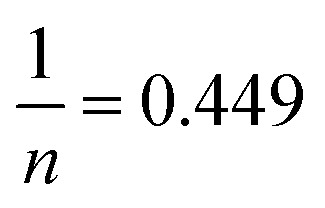	*k*_f_ = 3.27

The nature of the adsorption was addressed depending on the values of the dimensionless constant of Langmuir isotherm the dimensional constant known as the equilibrium parameter, *R*_L_ of Langmuir isotherm, and its value calculated from eqn (10):10
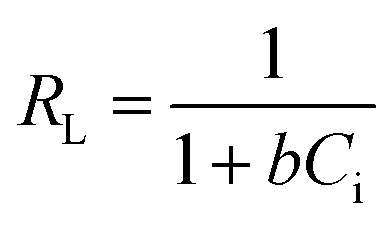
where *b* is a Langmuir constant and *C*_i_ is the initial concentration. The value of *R*_L_ indicates the nature of the adsorption process. *R*_L_ > 1, *R*_L_ = 1, 0 < *R*_L_ < 1, and *R*_L_ = 0 for unfavourable adsorption, linear adsorption, favorable adsorption, and irreversible adsorption, respectively.^15^ From this data, the parameter that show the *R*_L_ values for the removal of Cr(vi) ranged from 0.197 to 0.620 for GO. These values indicate favorable adsorption process for the GO. From the Freundlich isotherm model, the calculated value for (1/*n*) of adsorption Cr(vi) is less than 1, this refers to a heterogamous surface with minimum interactions between the adsorbent ions.

#### Thermodynamic parameter for adsorption process

The Gibbs free energy (Δ*G*°), entropy (Δ*S*°), and the enthalpy process (Δ*H*°) were calculated using the following van't Hoff eqn (11)–(13):11
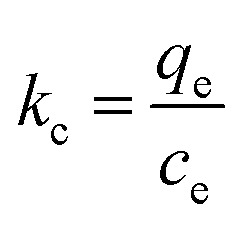

12 Δ*G*° = −*RT* ln *k*_c_

13
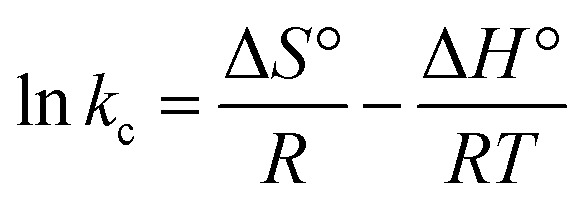
where *k*_c_ is the equilibrium constant calculated as the surface and solution metal distribution. Adsorption enthalpy and entropy were calculated from eqn (11) and the plot curve of ln *k*_c_*versus 1/T*, the values are presented in Table 3. The positive value of adsorption enthalpy shows that the process is endothermic, and its magnitude implies that the adsorption of Cr(vi) on GO is chemical adsorption.^4,16^ An increase in the equilibrium constant (*k*_c_) with the increase in the temperature also indicates an increase in the amount of the adsorbent metal ions.^20^

**Table:** Thermodynamic parameters for the adsorption of Cr(vi) onto GO-200 nm

*T* (K)	1/*T*	*k*_c_	ln *k*_c_	Δ*G*°	Δ*H*°	Δ*S*°
298	3.355 × 10^−3^	0.345	−1.062	2.631	4.30	5.56
318	3.144 × 10^−3^	0.377	−0.973	2.572		
328	3.048 × 10^−3^	0.407	−0.898	2.448		

Furthermore, the positive value of the adsorption entropy suggested increased randomness at the adsorbent-solution interface.^4,19^ The Δ*G*° can be calculated from eqn (14):

14 Δ*G*° = Δ*H*° − *T*Δ*S*°

which means the reaction is non-spontaneous at optimized conditions.

## Conclusions

In this study, the preparation of GO-200 nm for the removal of Cr(vi) under different experimental conditions was elucidated. The FTIR spectroscopy showed oxidation of graphite to GO and confirmed the formation of GO particles and GO/Cr(vi) interaction. Furthermore, the experimental results showed that the pseudo-second-order model and Langmuir isotherm model fitted well with the adsorption data. The thermodynamic parameter (Δ*G*°) indicated that the adsorption process is nonspontaneous.

## Data availability

We confirm that some of findings in this paper are available as part of master thesis of the first author Mrs Zohor Khdoor at Hebron University, Faculty of Graduate Studies, Chemistry Department. Here is the link: https://dspace.hebron.edu/jspui/bitstream/123456789/921.

## Conflicts of interest

The authors declare that no competing interests.
